# Cost and Effectiveness of Decontamination Strategies in Radiation Contaminated Areas in Fukushima in Regard to External Radiation Dose

**DOI:** 10.1371/journal.pone.0075308

**Published:** 2013-09-17

**Authors:** Tetsuo Yasutaka, Wataru Naito, Junko Nakanishi

**Affiliations:** 1 National Institute of Advanced Industrial Science and Technology (AIST), Institute for Geo-Resources and Environment (GREEN), Higashi, Tsukuba, Japan; 2 National Institute of Advanced Industrial Science and Technology (AIST), Research Institute of Science for Safety and Sustainability (RISS), Onogawa, Tsukuba, Japan; 3 National Institute of Advanced Industrial Science and Technology (AIST), AIST Fellow, Higashi, Tsukuba, Japan; Tel Aviv University, Israel

## Abstract

The objective of the present study is to evaluate the cost and effectiveness of decontamination strategies in the special decontamination areas in Fukushima in regard to external radiation dose. A geographical information system (GIS) was used to relate the predicted external dose in the affected areas to the number of potential inhabitants and the land use in the areas. A comprehensive review of the costs of various decontamination methods was conducted as part of the analysis. The results indicate that aerial decontamination in the special decontamination areas in Fukushima would be effective for reducing the air dose rate to the target level in a short period of time in some but not all of the areas. In a standard scenario, analysis of cost and effectiveness suggests that decontamination costs for agricultural areas account for approximately 80% of the total decontamination cost, of which approximately 60% is associated with storage. In addition, the costs of decontamination per person per unit area are estimated to vary greatly. Appropriate selection of decontamination methods may significantly decrease decontamination costs, allowing more meaningful decontamination in terms of the limited budget. Our analysis can help in examining the prioritization of decontamination areas from the viewpoints of cost and effectiveness in reducing the external dose. Decontamination strategies should be determined according to air dose rates and future land-use plans.

## Introduction

The accident at Tokyo Electric Power Company’s (TEPCO) Fukushima Dai-ichi Nuclear Power Plant (hereinafter referred to as F1NPP) in March 2011 released radionuclides into the environment and contaminated large areas of land in Japan. Approximately 22% of radionuclides released are thought to have been deposited on land [1]. Monitoring of large areas by aircraft has revealed a zone of high-density surface deposition of radionuclides (primarily caesium 134 and caesium 137) extending to the northwest from the nuclear power station [2]. The contaminated land area in Fukushima Prefecture with a potential annual air dose of 5 mSv extends for 1,778 km^2^ [3]. Due to concerns about the possibility of a large-scale release of radioactive materials and health risks, areas within a 20-km radius of the F1NPP (Restricted Area) and heavily contaminated areas outside of this zone (Deliberate Evacuation Area) have been designated. In days and weeks following the accident, approximately 85,000 people from 11 municipalities were evacuated from these evacuation areas, which cover approximately 1,170 km^2^ [3]. As of November 30, 2012, the Restricted Area and the Deliberate Evacuation Area have been rearranged into three areas: Area 1 (in which evacuation orders are ready to be lifted), Area 2 (in which residents are not permitted to live), and Area 3 (in which residents will face difficulties in returning for a long time) [4].

Following the accident, the Japanese government promulgated the Act on Special Measures Concerning the Handling of Environment Pollution by Radioactive Materials Discharged by the NPS Accident Associated with the Tohoku District: Off the Pacific Ocean Earthquake That Occurred on March 11 (Act on Special Measures Concerning the Handling of Pollution by Radioactive Materials) on December 8, 2011, with the goal of quickly reducing the impact of environmental pollution from the radioactive material on human health and the environment. Under this legislation, which was enacted January 1, 2012, a framework and guidelines for decontamination operations were released as Decontamination Guidelines (December 2011) [5], which covered methods for surveying and measuring the degree of contamination of the environment in intensely contaminated areas, as well as measures for decontamination and guidelines for collection, transport, and storage of removed soil. The Ministry of Environment also formulated a decontamination plan to be implemented under the direct supervision of the government and announced the Policy for Decontamination in the Special Decontamination Areas (Decontamination Road map; January 2012) [6].

Due to the societal interests and regulatory needs, numerous radiation monitoring or modelling studies to grasp and analyse the current situation in Fukushima have been conducted [7-9]. Pilot-scale investigations have also been conducted in order to develop efficient decontamination techniques and to evaluate their respective decontamination efficiencies [10,11]. These pilot-scale investigations revealed that the decontamination efficiency varies depending on land use or air dose rate, and even if decontamination is performed, the air dose rate cannot be reduced to the background radiation levels [12]. Most of the previous studies or reports regarding radiation contamination in Fukushima were rather reductionistic and focused primarily on determining the magnitude of contamination and the development or improvement of decontamination technologies. In order to establish effective and pragmatic decontamination methods for use in the radiation contaminated areas in Fukushima (primarily in the special decontamination areas), a holistic approach for assessing decontamination strategies, their costs, and long-term external radiation doses is needed.

For anthropogenic pollution, the polluter pays principle (PPP), in which the polluter is responsible for decontaminating and restoring the affected areas to their original states, should be applied. However, as mentioned above, the special decontamination areas alone span more than 1,100 km^2^, and the effectiveness of decontamination is limited, so that it is impossible to completely restore this area to its original state through decontamination in a short period of time.

Several studies have evaluated the effective dose and the effect of remediation of radioactive contamination in the case of the Chernobyl accident [13-16]. Most of these studies are retrospective, and analyses on the effects of decontamination were rather confirmatory. Yasutaka et al. [17] conducted a prospective quantitative evaluation of the reduction of the cumulated effective doses due to external radiation exposure for several decontamination scenarios using geographical information system (GIS) in special decontamination areas of Fukushima. However, the costs of the decontamination scenarios were not included in their analysis. Despite the enormous cost associated with radiation decontamination, almost no quantitative assessment has been performed on the relationship between the potential reduction in long-term radiation exposure and the costs of the various decontamination strategies considered for the special decontamination areas in Fukushima. There have been no studies to evaluate the efficiency of decontamination in a timely manner that consider the costs of multiple decontamination techniques.

The primary goal of our study is to provide quantitative information that is useful for decision-making in determining the prioritization of decontamination in the special decontamination area in Fukushima. The objective of the present study is evaluation of the costs and effectiveness of decontamination strategies in the special decontamination areas in Fukushima in regard to external radiation dose. GIS was used to relate the predicted external dose in the affected areas to the number of potential inhabitants and the land use in these areas. The choice of decontamination strategies or countermeasures in the special decontamination areas should be based on a comprehensive analysis of multiple attributes such as radiological, economic, and socio-psychological attributes. The cost and effectiveness of the different decontamination strategies is not the determinant of the remediation strategies of the special decontamination area but is one of the most important attributes when making the policy decision. In the present study, we focus on radiological and economic attributes in determining decontamination strategies.

## Materials and Methods

### Target area

**Figure 1:**
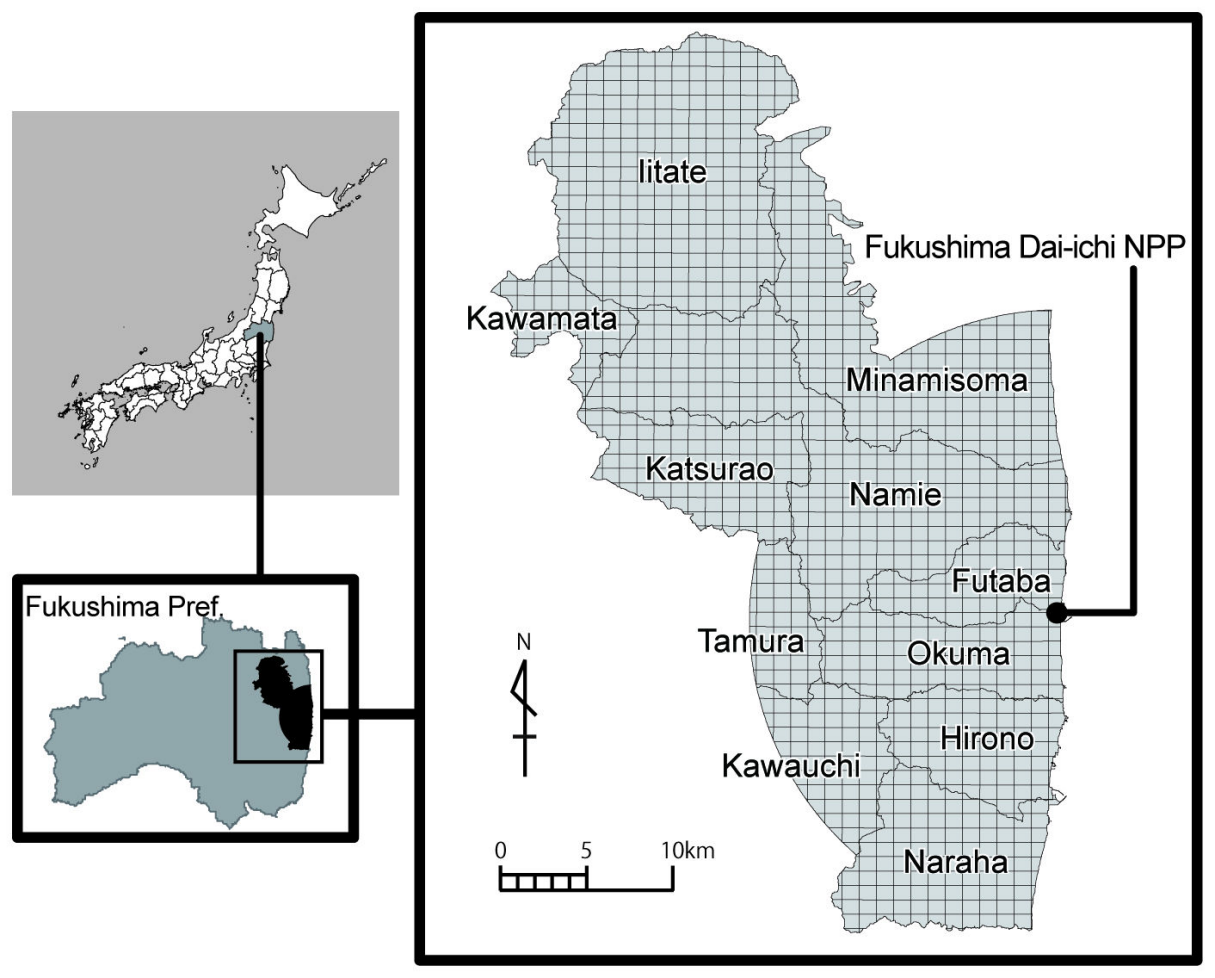
Target area of the present study.

The present study considers the contaminated areas from which people have been relocated after the accident. The target area of the present study is the special decontamination areas, where decontamination is being implemented by the national government. For this category, the “Restricted Area” (the area within a 20-km radius of the power station) and the “Deliberate Evacuation Area” (the area in which the annual cumulative dose could exceed 20 mSv) were designated in parts of 11 municipalities in eastern Fukushima Prefecture (Figure 1). Approximately 86,000 individuals were living in the study area as of year 2010 [18] (Table 1).

**Table 1:** General characteristics of the special decontamination areas.

	Special decontamination area
Population	86,274^a^
Area (km^2^)	1,142^b^
Land-use	Forest: 72%
	Agricultural: 20%
	Residential: 4%
	Others: 4%

^a^ Population is based on the 2010 Population Census [18]. The population in areas in which only the part of the municipality is affected was obtained by assuming the population to be proportional to the area.

^b^Estimated Based on GIS 1-km mesh data

### Air Dose Rate, Population Density, and Land Use

GIS is a powerful data integration and spatial analysis tool. In the present study, ArcGIS ver. 10.1 is used to aggregate, synthesize, and analyse large datasets and to identify spatial relationships among air dose rate, population density, and land use in the target area. Data on air dose rate (µSv/h) as of November 5, 2011 is obtained from MEXT [19] and the inverse distance weighted (IDW) approach was used to generate a continuous map. The data cover a range of within 80 km of the F1NPP and indicate the air dose rate distribution at a height of 1 m above the ground. For the decontamination efficiency analyses, 100-m-mesh data on the air dose rate were created using a continuous map. The air dose rate at the centre of each of the 100-m meshes was used as the representative value, and the arithmetic mean of the 100-m-mesh air dose rates was then used to calculate the air dose rate for each 1-km-mesh unit (mesh size: 1 km × 1 km). These values included a background level of radiation (average: 0.04–0.05 µSv/h) [20].

The 1-km-mesh population density data for the target area were obtained from the 2010 Population Census 2010 [18]. For the land-use data, we used 100-m-mesh data from national land numerical data provided by the Ministry of Land, Infrastructure, Transport and Tourism [21]. We reorganized the original 12 types of land use into six types: paddy fields, other agricultural land, forest, land for buildings, roads, and other uses. In the following analysis, we consider paddy field and other agricultural land as agricultural areas, land for buildings as residential and building areas, roads as road and street areas. We do not consider other uses (e.g., lakes and rivers, waste lands) as the subject of decontamination.

For the total road length, we used the road density and total road length mesh data from the national geographical numerical data [21]. These data comprise the total extended road distance by road width for each 1-km mesh.

### Estimation of external dose

Radiation exposure may be internal or external and can be acquired through various exposure pathways. Dose estimation models that consider both types of pathways can be found elsewhere [14,15]. Since the external dose due to deposited radionuclides is a main pathway of the total radiation and the classification of decontamination areas is based solely on the additional external dose [22], only external exposure is considered in the present study.

The additional annual external dose, *D*_*ext,i*_ [mSv], at location *i*, is calculated as follows:

$$D_{ext,i}=(p_{i}-p_{nat})\cdot (T_{out}+T_{in}\cdot \beta )\cdot 365/1000$$ (1)

where *p*_*i*_ is the air dose rate [µSv/h] at location *i*, *p*_*nat*_ is the air dose rate [µSv/h] from naturally occurring radionuclides, *T*_*out*_ is time spent outdoors [hours/day] at location *i*, *T*_*in*_ is the time spent indoors [hours/day] at location *i*, and β is the shielding factor, which is generally expressed as the ratio of the on-site external radiation level indoors to the on-site external radiation level outdoors. This simple equation is used by the central government of Japan to classify decontamination areas [22] under the Act on Special Measures Concerning the Handling of Pollution by Radioactive Materials. In order to estimate the annual external dose, the value of *p*_*nat*_ was found to be 0.04 [µSv/h], the indoor *T*_*in*_ and outdoor *T*_*out*_ dwell times were set to 16 hours and 8 hours, respectively, and the shielding factor was set as 0.4. These are the same values used by the central government [22]. In a very simple expression, the additional dose from external irradiation is calculated by the absorbed dose in air multiplied by a reduction factor of 0.6. Evidence has indicated that the additional dose from external irradiation may be well below the estimated additional dose calculated by the simple model [11]. Realistic dose estimations from external irradiation exposure can be an important issue. Since a full discussion of this issue is beyond the scope of the present study, in order to be consistent with the existing regulatory approach, we followed the assumption of the central government in the present study, unless otherwise stated.

### Decontamination methods, efficiency, and cost

Appropriate selection of decontamination strategies during the rehabilitation stage of radiation-affected areas can significantly affect decontamination costs. The costs of decontamination, which depends on the land use of the areas, can be classified as 1) clean-up costs and 2) storage costs. The clean-up costs include the cost of removal or reduction for contaminated materials and soil, containers for temporary storage, and temporary storage, while the storage costs include the cost of volume reduction, interim storage, and final disposal. Since no information is available regarding the cost of final disposal, this cost was not considered in the present study. The decontamination methods and their respective unit costs for the various land uses considered in the present study are presented in Table 2 and Table 3. Cost data for various decontamination methods were based primarily on data from previous studies [10,11]. A comprehensive report was published by JAEA [11] on different decontamination methods based on a large-scale pilot study conducted in Fukushima in 2011 and 2012. Estimating the cost of interim storage is difficult because there has been no decision as to whether to implement volume reduction or where or how to go about volume reduction of the contaminated waste and soil generated due to clean up. In order to estimate the magnitude of the interim storage cost, however, we have attempted to estimate the cost of interim storage based on the unit cost of the existing controlled or shielded landfill. Combustible waste was assumed to subject to volume reduction. Moreover, the indirect costs and the costs of radiation control were not considered in the present study. Due to a lack of information, the general administrative expenses and the cost related to decreasing working efficiency in high-air-dose-rate areas were not considered in the present study.

**Table 2:** Unit cost and number of flexible containers for clean-up phase of decontamination for different method considered in the present analysis.

			Unit cost (10 thousand JPY/hectare)			Number of flexible containers ^a^ (units/hectare)	
Land-use	Abbr.	Decontamination Option	Removal	Flex. containers	Temporary storage ^b^	Incombustible	Combustible
Agricultural	A1	Weeding/Stripping 5cm topsoil/Covering Soil	950^c^	652	1,630	715^d^	100^d^
	A2	Weeding/ Stripping 5cm topsoil	625^d^	652	1,630	715^d^	100^d^
	A3	Interchanging topsoil with subsoil	310^d^	0	0	0	0
	A4	Plowing	33^d^	0	0	0	0
Forest	F1	Removal of fallen leaves and humus surface	745^d^	424	1,060	270^d^	260^d^
Residential and building	RB1	Whole decontamination	1750^c^	120	300	140^d^	0
Road and street	RS1	Shot blasting	480^d^	24	60	30^d^	0

^a^Number of flexible containers used to store contaminated wastes generated by removal phase. The volume of a container is assumed to be 0.9 m^3^ and the cost of a flexible container is assumed to be 8,000 JPY/unit.

^b^The cost of the temporary storage per flexible container is assumed to be 20,000 JPY/unit.

^c^ Obtained from Fukushima [10].

^d^ Obtained from JAEA [11]. For the decontamination cost for F1, the unit cost was estimated considering the slope angle of the area based on the report.

**Table 3:** Unit cost for storage phase of decontamination and total of all the unit costs considered in the present analysis.

			Unit cost (10 thousand JPY/hectare)					
			Interim storage ^a^			Total ^b^		
Land-use	Abbr.	Decontamination Option	D1	D2	D3	D1	D2	D3
Agricultural	A1	Weeding/ Stripping 5cm topsoil/Covering Soil	2,215	7,220	8,150	5,447	10,452	11,382
	A2	Weeding/ Stripping 5cm topsoil	2,215	7,220	8,150	5,122	10,127	11,057
	A3	Interchanging topsoil with subsoil	0	0	0	310	310	310
	A4	Plowing	0	0	0	33	33	33
Forest	F1	Removal of fallen leaves and humus surface	992	2,882	5,300	3,221	5,111	7,529
Residential and building	RB1	Whole decontamination	450	1,500	1,500	2,620	3,670	3,670
Road and street	RS1	Shot blasting	90	300	300	654	864	864

^a^Three options are assumed for the interim storage: D1 assumes combustible waste to be subjected to volume reduction and different types of disposal for high-elution materials (isolation-type disposal) and low-elution materials (control-type disposal) are used. D2 assumes combustible waste to be subjected to volume reduction and isolation-type disposal for both high-elution and low-elution materials is used. D3 assumes no volume reduction and isolation-type disposal is used for all waste.

^b^Total consists of clean-up and storage costs.

The efficiency of decontamination depends on the air dose rate and land use [17]. The values of decontamination efficiency (*φ*) for different land use and air dose rates considered in the present study are shown in Table 4. No data are available for the decontamination efficiencies for the air dose rates for method A3 (interchanging topsoil with subsoil). Therefore, we have obtained data from JAEA [23] and the same ratio of the reduction factors for methods A1 and A2 are temporality used for methods A3 and A4.

**Table 4:** Decontamination efficiencies for different decontamination methods [17].

		Decontamination efficiency ^a^ (*φ*)			
Land-use	Abbr.	< 1 µSv/h	1-3 µSv/h	3-10 µSv/h	> 10 µSv/h
Agricultural	A1	0.34	0.49	0.47	0.80
	A2	0.34	0.49	0.47	0.80
	A3	0.34	0.49	0.47	0.80
	A4	0.21	0.31	0.29	0.50
Forest	F1	0.19	0.27	0.39	0.59
Residential and building	RB1	0.29	0.35	0.49	0.70
Road and street	RS1	0.15	0.30	0.41	0.66

^a^The values of the decontamination efficiency were estimated based on data on the efficiency of the aerial decontamination in the municipalities.

### Calculation of the air dose rate in each 1-km mesh with decontamination

Forest adjacent to 100-m-mesh units of paddy fields, other agricultural land, or buildings were assumed to be decontaminated, whereas other areas of forest were assumed not to be decontaminated. Moreover, of the mesh units that contained forest to be decontaminated, we assumed that only 20% of the forests in these mesh units would be decontaminated, as specified in the guidelines of the Ministry of the Environment [5].

The air dose rate with decontamination was calculated by multiplying the 100-m-mesh air dose rate (excluding background value) without decontamination by the residual ratio (= 1-*φ*). The estimated air dose rates with decontamination for the 100-m-mesh units of land-use categories subject to decontamination (land for buildings, paddy fields, other agricultural land, roads, and decontaminated forest) within each 1-km-mesh unit were averaged, and these results were considered to be the air dose rates with decontamination for the respective 1-km-mesh units. If there were no land-use categories subject to decontamination within a given 1-km-mesh unit, then the air dose rate was taken to be equal to the air dose rate without decontamination.

### Calculation of prospective external dose

In order to evaluate long-term additional external dose, the followings are the major assumptions for the estimation model: 1) the concentration ratio of ^134^Cs and ^137^Cs on March 25, 2011 was 1:1, 2) the air dose rate on November 5, 2011 was attributable only to ^134^Cs and ^137^Cs, 3) the reductions in concentration of ^134^Cs and ^137^Cs was due only to physical decay, and 4) the return of all evacuated residents to the special decontamination areas would be on April 1, 2014 (three years after the accident). Specifically, assuming the return of all former residents on April 1, 2014, we calculated the long-term additional external dose from that date. The parameters, including the half-life of radioactive Cs (T_134_Cs__= 2.1 years; T_137_Cs__= 30.0 years) and the conversion coefficient CF3 peripheral air dose rate from deposition (5.4E-6 [(mSv/h)/(kBq/m^2^)] for ^134^Cs and 2.1E-6 [(mSv/h)/(kBq/m2)] for ^137^Cs) were obtained from IAEA [24] and were used to estimate the contribution ratio of each nuclide to the air dose rate. As of November 5, 2011, the contribution ratios of ^134^Cs and ^137^Cs were estimated to be 0.69 and 0.31, respectively.

The air dose rate [mSv/y] *t* years after November 5, 2011 at location *i*, *D*_*i*_ (t), is calculated according as follows:

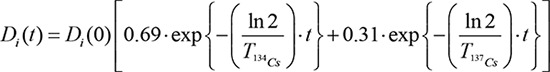
(2)
where *D*_*i*_(0) is the air dose rate [mSv/y] as of November 5, 2011 at location *i*.

The air dose rate [mSv/y] at location *i* as of April 1, 2014, *D*_*i,Apr.1_2014*_, is calculated as follows:


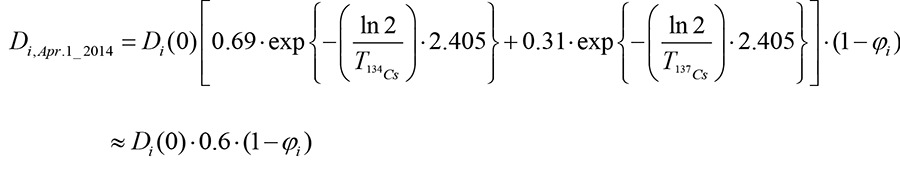
(3)

where 2.405 is the period between November 5, 2011 and April 1, 2014 expressed in years, *φ_i_* represents the efficiency of the decontamination operation at location *i* expressed as the ratio of the air dose rate with decontamination to the air dose rate without decontamination, which is zero when no decontamination operation is performed.

### Decontamination scenario

Seven decontamination scenarios (SC) are considered in the present study:

SC1: A1+F1+RB1+RS1 with D1
SC2: A1+F1+RB1+RS1 with D2
SC3: A1+F1+RB1+RS1 with D3
SC4: A2+F1+RB1+RS1 with D1
SC5: A3+F1+RB1+RS1 with D1
SC6: A4+F1+RB1+RS1 with D1
SC7: A1+F1+RB1+RS1 with D1 and the full decontamination of the forest areas

Of the above scenarios, we assume SC1 to be the basic scenario in the present study because this scenario includes the standard decontamination methods for each land use described in the previous report [11]. For each land use, the decontamination options are as follows: for agricultural areas, A1 involves weeding, stripping 5 cm of topsoil, and covering soil; A2 involves weeding and stripping 5 cm of topsoil; A3 involves interchanging top soils with subsoil; A4 involves ploughing only. The decontamination option of forest areas (F1) involves the removal of fallen leaves and humus surface. Whole-area decontamination is a typical option for residential and building areas (RB1) and involves washing or wiping off house walls and roofs, and the removal of weeds, topsoil, and plants in gardens. Shot blasting was assumed to be a typical option for roads and street areas (RS1). With respect to the options for interim storage, we assumed three different options: D1, D2, and D3. D1 is the basic option, which assumes combustible waste to be subjected to volume reduction and different types of disposal for high-elution materials (isolation-type disposal) and low-elution materials (control-type disposal) are used. For D2, combustible waste is assumed to be subjected to volume reduction and isolation-type disposal for both high-elution and low-elution materials. For D3, no volume reduction is assumed, and isolation-type disposal is used for all waste. Among SC1, SC2, and SC3, the impacts of the choice of the interim storage methods are examined, and the effects of the choice of SC1, SC4, SC5, or SC6 in the agricultural areas on the total decontamination cost are examined. The effects of decontamination methods in agricultural and forest areas on external dose are examined using SC1, SC6, and SC7.

## Results and Discussion

### Spatial relationships among air dose rate, population density, and land use with or without decontamination

**Figure 2:**
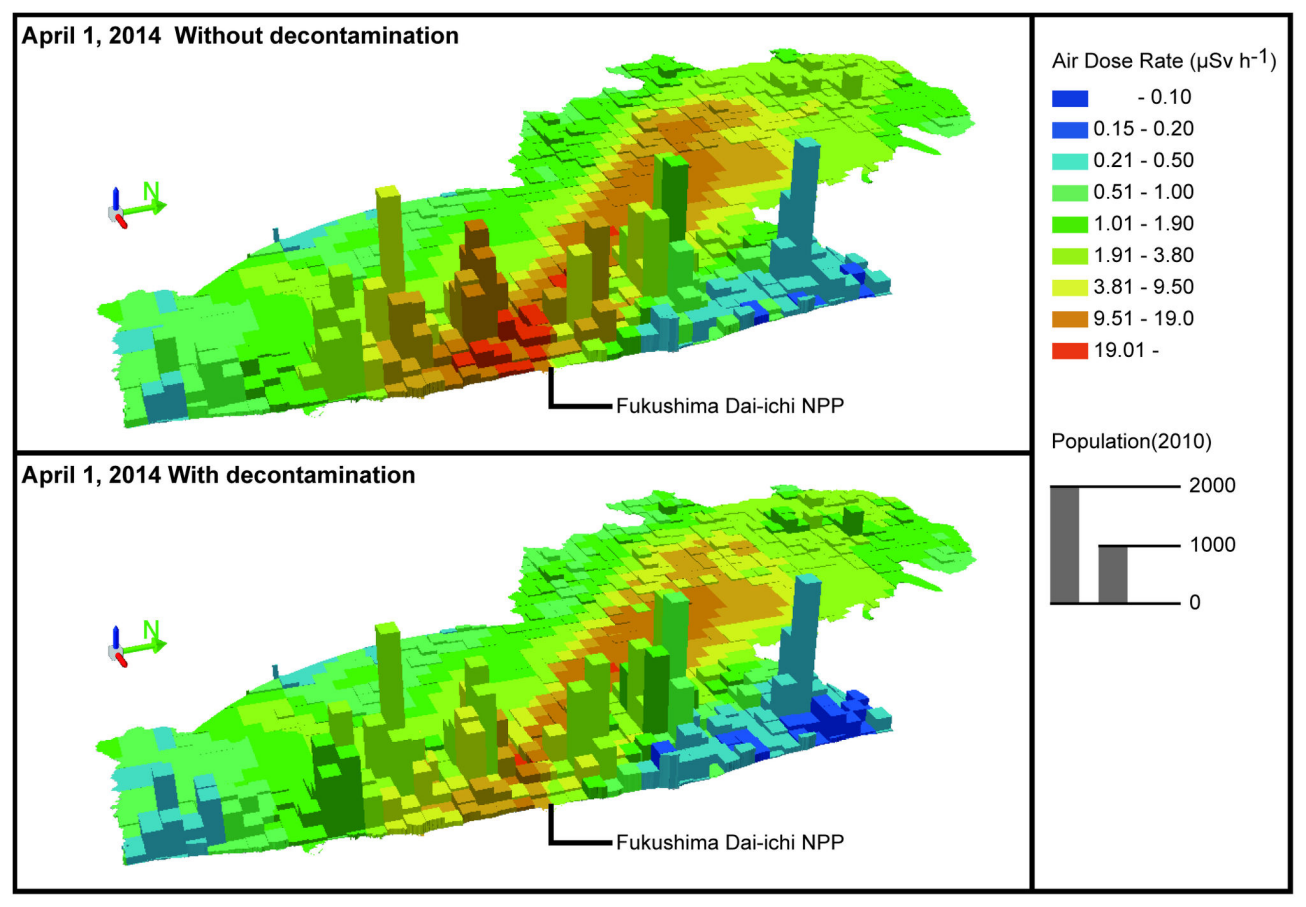
Estimated air dose rate for the special decontamination areas in Fukushima as of April 1, 2014. Population data are based on the 2010 Population Census.

**Figure 3:**
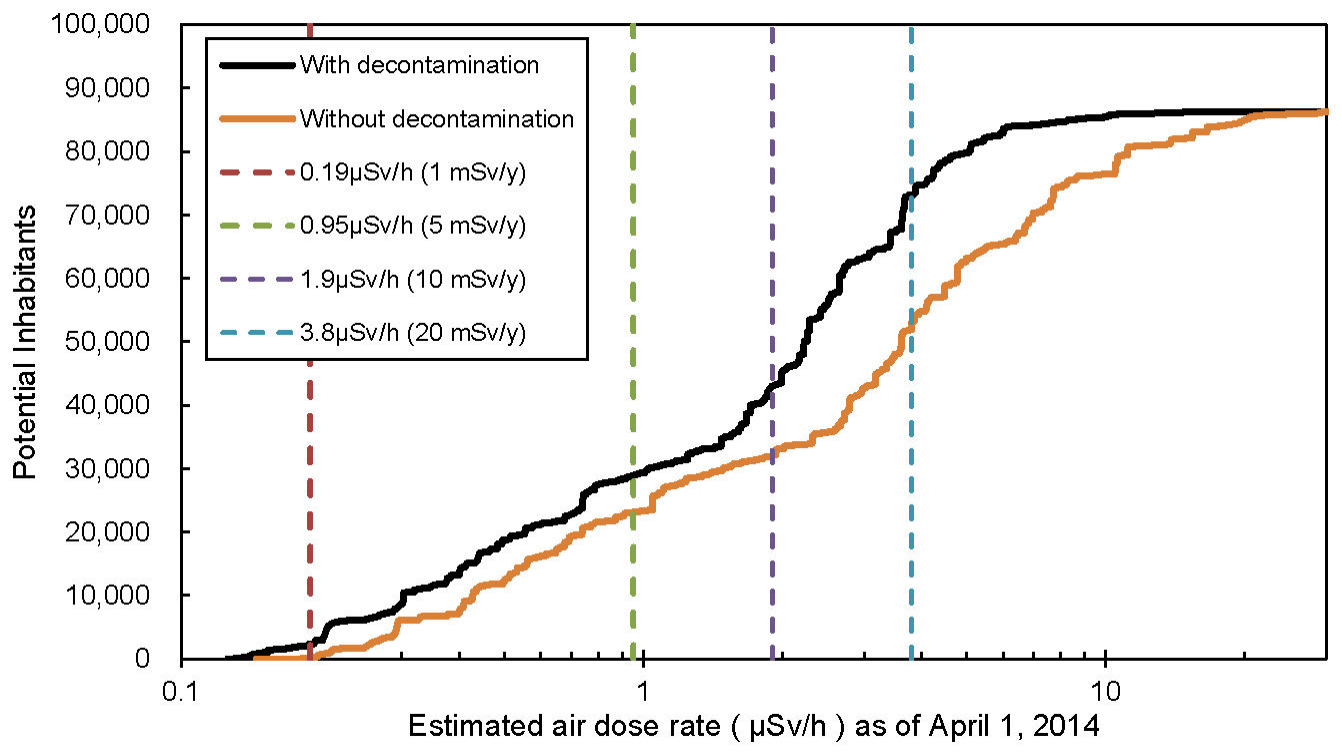
Relationship between the cumulated potential inhabitants and the estimated air dose rate in the special decontamination areas in Fukushima as of April 1, 2014. The cumulated potential inhabitants are based on the 2010 Population Census.

Figure 2 shows the spatial relationships among the estimated air dose rate, potential population density, and land use with or without decontamination in the special decontamination areas as of April 1, 2014 for SC1. Approximately more than 70% of land is covered by forest, and coastal regions have high population density. Assuming that the forest in the vicinity of the residential area is the target of decontamination, the potential decontamination area of the present study covers an area of approximately 437 km^2^. According to the official estimation equation of external dose, within this potential decontamination area, the areas in which the annual external dose is estimated to exceed 1 mSv (air dose rate: approximately 0.19 µSv/h), 5 mSv (air dose rate: approximately 0.95 µSv/h), and 20 mSv (air dose rate: approximately 3.8 µSv/h) on April 1, 2014 would cover 277 km^2^, 196 km^2^, and 55 km^2^, respectively. Without decontamination, the area exceeding an annual external dose of 1 mSv was estimated to cover 288 km^2^, the area exceeding an annual external dose of 5 mSv was estimated to cover 222 km^2^, and the area exceeding an annual external dose of 20 mSv was estimated to cover 94 km^2^. With decontamination, approximately 2,300, 28,900, and 73,100 potential inhabitants were estimated to be returnable for the target levels of 1 mSv, 5 mSv, and 20 mSv, respectively (Figure 3). Approximately 30,000 potential inhabitants would live in areas of relatively low contamination with annual external doses of less than 5 mSv with decontamination. The 1-km-mesh of the highest population density, which is located in Tomioka Town, would have estimated annual external doses of 25 mSv (air dose rate: approximately 4.8 µSv/h) without decontamination and 14 mSv (air dose rate: approximately 2.7 µSv/h) with decontamination on April 1, 2014. These results indicate that aerial decontamination in the special decontamination area would be effective in reducing the air dose rate to the target levels for some but not all of the areas.

A reduction factor of 0.6 was used in the official equation for estimating external dose [22]. External doses estimated using a reduction factor of 0.6 may be conservative and may overestimate the external effective doses in the affected area. In the external dose estimation at Chernobyl, the reduction factors ranged from 0.20 to 0.36, depending on the ages and occupations of individuals [25]. Yasutaka et al. [17] revealed that the selection of reduction factor in estimating external doses is important to determine the area of decontamination. In the post-emergency phase, as knowledge and information regarding external dose in Fukushima have been accumulated, careful consideration of the reduction factor should be incorporated into the examination of future remediation plans of the affected areas.

### Estimated cost of decontamination in the special decontamination areas

The estimated costs of different decontamination methods for various land uses and the estimated costs for different decontamination scenarios in the special decontamination areas in Fukushima are shown in Table 5 and Table 6, respectively. Assuming the standard decontamination scenario, SC1, the estimated total cost of decontamination is approximately 1,300 billion JPY. Based on the estimation result, the decontamination costs for agricultural land were estimated to be more than 1,000 billion JPY, which account for approximately 80% of the total decontamination cost. Of the total cost, the percentages of decontamination costs in SC1 are estimated to be approximately 9% for forest areas, 8% for residential areas, and less than 1% for roads and streets. Of the total decontamination cost for agricultural land, the cost of storage is estimated to be 60%. For SC2 and SC3, the estimated cost of interim storage varies widely depending on the requirements of the interim storage. As an alternative to soil removal, which requires storage of a large volume of removed soil, either deep plowing or reversal tillage can be used to decontaminate agricultural land, making it possible to significantly reduce the cost of removed soil storage. According to the cost estimations for SC4, SC5, and SC6, the cost of decontamination in agricultural land would decrease by 99% in the case of using reversal tillage. Our calculation suggests that the appropriate selection of a decontamination method would significantly decrease the cost of decontamination. Note that if the plough pan is shallow, ploughing or reversal tillage cannot be applied because it may cause subsurface leakage of water, resulting in useless paddy fields. Assuming full decontamination of the forest areas (SC7), which seems unlikely, the estimated cost of decontamination would rise significantly.

**Table 5:** Estimated costs of different decontamination methods for various land uses in the special decontamination areas in Fukushima.

		Estimated cost (billion JPY)								
					Interim storage			Total		
Land-use	Abbr.	Removal	Temporary storage	Flex. Containers	D1	D2	D3	D1	D2	D3
Agricultural	A1	190	325	130	442	1,440	1,626	1,086	2,085	2,270
	A2	125	325	130	442	1,440	1,626	1,022	2,020	2,205
	A3	62	0	0	0	0	0	62	62	62
	A4	7	0	0	0	0	0	7	7	7
Forest	F1	25	40	16	38	109	201	119	190	282
Residential and building	RB1	70	12	5	18	60	60	105	147	147
Road and street	RS1	6	0.7	0.3	1	4	4	8	10	10

**Table 6:** Estimated costs for different decontamination scenarios.

		Estimated cost (billion JPY)				
Decontamination Scenario		Removal	Temporary storage	Flex. Container	Interim storage	Total
SC1	A1+F1+RB1+RS1 with D1	290	378	151	499	1,318
SC2	A1+F1+RB1+RS1 with D2	290	378	151	1,613	2,432
SC3	A1+F1+RB1+RS1 with D3	290	378	151	1,891	2,710
SC4	A2+F1+RB1+RS1 with D1	225	378	151	499	1,253
SC5	A3+F1+RB1+RS1 with D1	162	53	21	57	293
SC6	A4+F1+RB1+RS1 with D1	107	53	21	57	238
SC7	A1+F1+RB1+RS1 with D1	817	1,245	498	1,310	3,870

For decontamination efforts under the Act on Special Measures Concerning the Handling of Pollution by Radioactive Materials, more than 1,500 billion JPY has been set aside to date [26] and additional budgetary requests are expected. The rationale of the proposed costs by the central government is unclear. Compared with the cost of the decontamination effort proposed by the government, the results of the presents study indicate that the proposed costs are underestimated. The cost of decontamination would increase significantly if the Intensive Contamination Survey Area is included in the estimation. In addition, the IAEA [24] criticized the plan to remove large volumes of low-radiation topsoil and waste for storage at secure facilities for an extended period. According to the IAEA [24], this could create unnecessary major challenges without providing any benefit in terms of reducing the radiation dosage. Even so, the total cost of decontamination would vary significantly depending on which decontamination strategy is selected, especially in the case of agricultural land.

**Figure 4:**
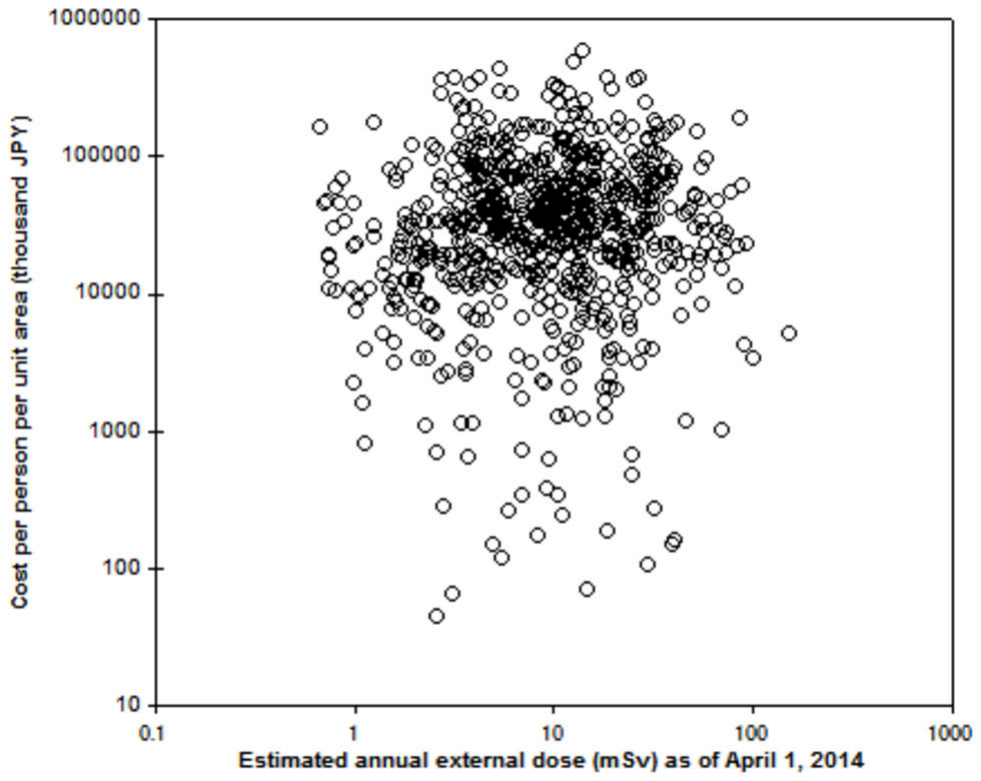
Relationship between the estimated cost of decontamination per person per unit area and the estimated annual external dose with decontamination in the special decontamination areas in Fukushima. Cost per person per unit area was calculated by the estimated decontamination cost divided by the population of the unit area. Population data are based on the 2010 Population Census.

The costs of decontamination per person per unit area (1 km^2^) were estimated for SC1 (Figure 4). The cost was estimated to range between 10 thousand JPY/person and 1 billion JPY/person. The geometric mean of the costs of decontamination per person per unit area was estimated to be approximately 28 million JPY. Approximately 14% of unit areas were estimated to require greater than 100 million JPY/person to conduct decontamination. Our analysis suggests that the cost of decontamination per person for each unit area varies greatly depending on population density and the type of land use. Compared with urban areas having high population densities, agricultural areas would have higher decontamination costs per person. If the objective of decontamination is to maximize population living in the affected areas after decontamination, which could be a cost-effective strategy, then special consideration should be given to the decontamination of urban residential areas. As shown in the previous section, assuming SC1 is implemented, the decontamination costs for agricultural areas are high and the efficiency of the decontamination is limited to a certain degree. Although restoring the original state of an area by decontamination can be a basic philosophy for large-scale anthropogenic pollution, it is important to understand the limitations of current decontamination techniques. As such, the decontamination strategies and areas should be prioritised in terms of cost, effectiveness, land use, and radiological protection (external dose exposure) after decontamination. Complete decontamination of the area cannot be the ultimate goal, and decontamination strategies should be examined considering the rehabilitation planning of the affected areas. However, land use for food production and the resulting internal doses, which were not considered herein, may modify this analysis.

### Long-term trends in the potential inhabitants and areas after decontamination

**Figure 5:**
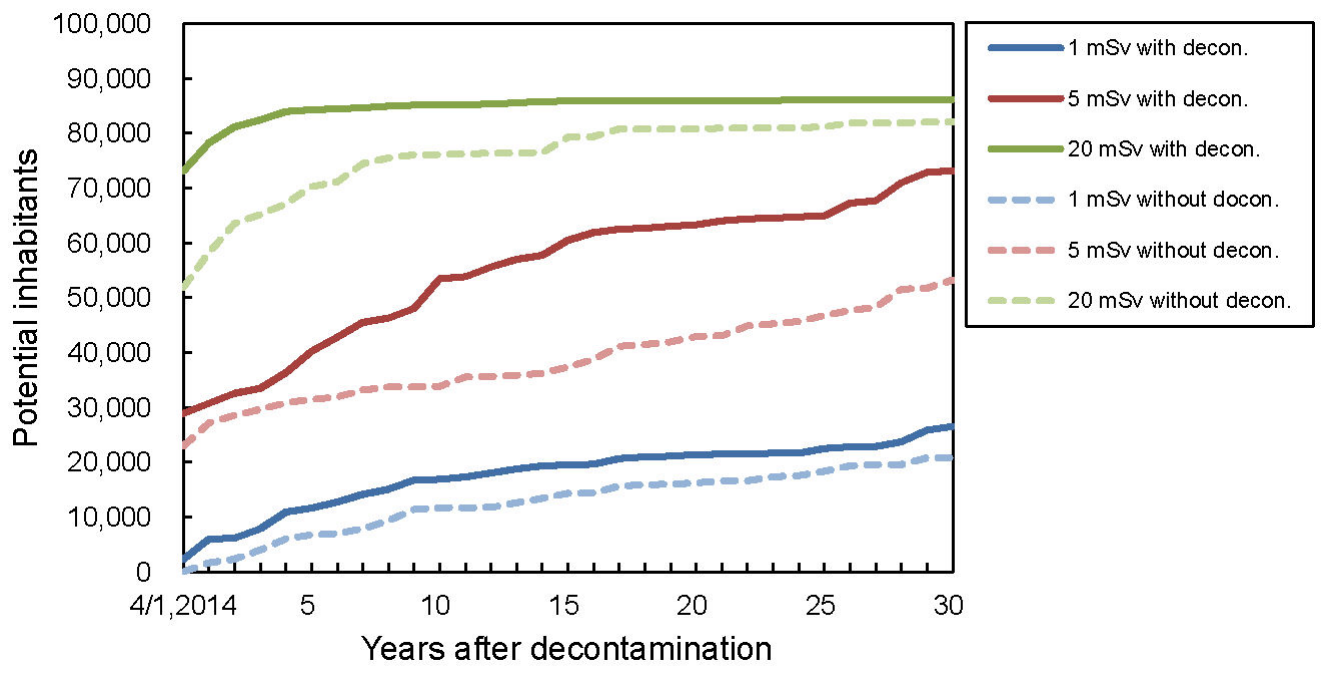
Estimated trends of potential inhabitants returning to their former residence areas for different dose levels with and without decontamination. Population data are based on the 2010 Population Census, and demographic factors were not considered in this analysis.

Figure 5 shows the trends of potential inhabitants returning to their former residence areas for different dose levels with and without decontamination. According to prospective calculation, in the year 2019, the numbers of the potential inhabitants with annual doses below 1 mSv, 5 mSv, and 20 mSv increase to approximately 11,600, 40,300, and 84,300, respectively with decontamination. In the year 2029, the number of potential inhabitants with annual doses below 1 mSv, 5 mSv, and 20 mSv are estimated to be 19,500, 60,500, and 85,900, respectively.

The majority of the potential inhabitants are estimated to be able to return to their former areas of residence 5 years after the completion of the aerial decontamination if the target level is set to 20 mSv/y and the public attitude toward returning home is ignored. If the target level is set to 5 mSv/y, a distinct difference in the number of potential inhabitants with and without decontamination was predicted after several years of the proposed completion of decontamination. The figure also shows that setting the target level to 1 mSv/y results in a small difference between the number of potential inhabitants with and without decontamination, and approximately 20,000 potential inhabitants are expected to live in the affected areas, even 30 years after the proposed completion of decontamination. Note that all of the predictions of external dose in the present study were based on the results of the Fourth Airborne Monitoring Survey by MEXT [19] assuming only natural radioactive decay and ignoring realistic occupancy/shielding factors. The prediction may be conservative and may overestimate the actual external dose in the affected area. In addition, we used the demographical statistics for 2010 to estimate the number of potential inhabitants returning to their former residences. This tends to overestimate the number of potential inhabitants returning of their former residence. Many of the former residence may not return to their former homes even after decontamination.

## Conclusions

The present analysis of the cost and effectiveness of decontamination in the special decontamination areas in Fukushima has revealed that the selection of decontamination methods would result in a significant difference in the costs of decontamination. Special attention should be paid to decontamination methods in agricultural areas because decontamination methods are the central element in estimating total cost in the special decontamination areas. In addition, the costs per person per unit area are estimated to vary greatly. The present study has revealed that aerial decontamination in the special decontamination area would be effective for reducing the air dose rate by the target level in a short period of time for some but not all of the area. We focused on radiological external dose and economic cost attributes in determining decontamination strategies using data that were available as of December 2012. The assumptions and model used to estimate external dose in the present study were chosen following those used by the central government, which do not match the actual external doses of individuals expected in the affected area. Monitoring of air dose rates and estimation of personal dose in Fukushima have been continued and scientific understanding of behaviour and exposure of radionuclides may improve. The values presented here should be regarded as estimations of the magnitude of the cost and effectiveness of the decontamination strategies in the special decontamination areas in Fukushima. Thus, further data and evidence may be used to adjust and refine the analysis.

The cost and effectiveness of the different decontamination strategies is not the sole determinant of the remediation strategies of the special decontamination area, but is one of the most important attributes when developing remediation strategies. Oughton et al. [27] pointed out that the evaluation of remediation measures must address both the social and ethical costs in addition to simple cost–benefit effectiveness. Therefore, it is important to assess not only the cost and effectiveness of decontamination, but also the social and ethical aspects of the decontamination strategies. Public acceptance, the willingness of inhabitants returning to their former area of residence, and social sustainability after decontamination during the rehabilitation planning phase must also be addressed in order to ensure the long-term sustainability of the affected area. The results of the present study can help in the prioritization of decontamination areas from the viewpoints of cost and effectiveness in reducing the external dose. In order to make the most of a limited decontamination budget, decontamination strategies should be determined according to the air dose rates and future land-use plan. In addition, for residents returning to restricted areas, it is important to evaluate long-term external radiation doses after the decontamination. Moreover, in order for affected municipalities to establish a sustainable society in the future, human factors, such as the inclination of people to return to their hometowns, are indispensable.
